# Mortality Due to Complications Associated With Acute Ogilvie’s Syndrome in an Older Adult Treated for Psychosis: A Case Report

**DOI:** 10.7759/cureus.51389

**Published:** 2023-12-31

**Authors:** Maryam M Ali, Mahmood Al Saeed, Mohamed Ebrahim, Fatima Mandeel

**Affiliations:** 1 Department of Internal Medicine, Salmaniya Medical Complex, Manama, BHR; 2 Department of Radiology, Salmaniya Medical Complex, Manama, BHR

**Keywords:** delusional disorder., antipsychotics, intestinal perforation, intestinal obstruction, ogilvie's syndrome, acute intestinal pseudo-obstruction

## Abstract

Acute colonic pseudo-obstruction or Ogilvie's syndrome is a disorder causing massive colonic dilation with no evidence of mechanical obstruction. The actual incidence of acute colonic pseudo-obstruction is unclear; However, electrolyte imbalance, psychiatric disorders, the use of medications such as anticholinergics or antipsychotics, and recent abdominal surgery are the most common predisposing factors associated with this syndrome. Ogilvie's syndrome is most likely caused due to impairment of the gut's motor system and an imbalance of the autonomic nervous system including a reduction in the activity of stimulatory neurotransmitters. The predisposition to psychotic disorders could be, in some instances, due to neurodevelopmental abnormalities of the brain and the gut’s autonomic nervous system. The symptoms of Ogilvie's syndrome are similar to mechanical obstruction of the colon but no physical cause of obstruction is usually present. Ogilvie's syndrome can be managed conservatively; however, if left untreated, Ogilvie's syndrome can lead to bowel perforation, which is associated with a high mortality risk. Antipsychotics have been considered the cornerstone treatment for psychiatric disorders including schizophrenia. Even though they are highly effective in treating psychiatric illnesses, their usage carries multiple risks. Overall, constipation is a common side effect of antipsychotic medications with some classes posing more risk than others. Constipation can be severe and may lead to serious complications such as paralytic ileus, bowel ischemia, and death. We present here a case of delusional disorder managed with risperidone and complicated by intestinal pseudo-obstruction. This case reiterates the need to consider all complications of antipsychotic medications, even rare ones, and include them in the discussion with patients and their caregivers before commencement.

## Introduction

Ogilvie's syndrome is a clinical condition characterized by abdominal distention without mechanical obstruction. Abdominal distention and bloating, abdominal pain, nausea, and vomiting are common symptoms of Ogilvie’s syndrome [1]. In some cases, the condition can be chronic and lead to prolonged periods of severe constipation [1]. Multiple risk factors for the development of Ogilvie's syndrome have been described, including electrolyte imbalances, advanced age, immobility, and poor functional status [2]. The general consensus regarding the pathogenesis of Ogilvie's syndrome is that the motor impairment of the gut is due to the dysfunction or imbalance of the autonomic nervous system [2].

Antipsychotics are a type of psychotropic medication used mainly to treat psychosis, especially in schizophrenia [3]. They are licensed to treat certain types of mental health problems when symptoms include psychotic experiences. Antipsychotic-related constipation is a common and serious adverse effect, especially with clozapine [4]. One of the most common serious adverse effects of clozapine is gastrointestinal hypomotility or “slow-gut” [5]. Clozapine-induced gut hypomotility can lead to paralytic ileus, bowel obstruction, gastrointestinal ischemia, toxic megacolon, and death [5]. On the other hand, the literature rarely reports cases of intestinal pseudo-obstruction due to risperidone [6].

The distinction between Ogilvie’s syndrome and mechanical obstruction may be difficult to identify based solely on signs and symptoms [1]. A radiographic examination of the colon is therefore usually performed to rule out a site of mechanical obstruction. Plain abdominal radiography can reveal an abnormally dilated colon. Additionally, plain radiographs can also reveal dilatation and abnormal air-fluid levels in the small bowel, which are both indicative of intestinal obstruction [1]. Perhaps, the most important use of plain radiography is to measure the degree of initial colonic dilatation and for serial imaging during follow-up observation after the diagnosis is made. CT scanning with oral and IV contrast is the preferred modality for diagnosis [2]. Rectal contrast can be helpful but has been linked to iatrogenic perforation [2]. Gastrografin or other water-soluble enteral contrasts are the preferred choice [7]. CT scanning will rule out anatomic or mechanical bowel obstruction as well as evaluate for more obscure causes of dilatation, such as retroperitoneal hematoma or abdominal abscess. CT scans can also identify signs of ischemia, such as mucosal wall thickening, submucosal edema, or gas [2]. Classically, acute colonic pseudo-obstruction (ACPO) on CT scan will show isolated dilatation of the cecum and ascending colon and a transition zone at the splenic flexure that is gradual [2]. Treatment involves correction of the underlying condition including any biochemical abnormalities [1]. Medical treatment options include anticholinesterases like neostigmine and antibiotics such as erythromycin [1]. Complications from Ogilvie’s syndrome can be fatal and carry high mortality rates between 35% and 72% [8]. These complications include intestinal perforation and bowel ischemia.

Here, we present the case of a patient whose death was likely attributed to complications associated with Ogilvie’s syndrome. This was likely related to antipsychotic medication use, which reiterates the importance of awareness of this association, early diagnosis, and timely clinical management to prevent morbidity and mortality.

## Case presentation

A 78-year-old man presented to our emergency department with abdominal pain and poor appetite. He had a background medical history of hypertension, diabetes mellitus, and a newly diagnosed delusional disorder. The patient has been following up in the outpatient psychiatry clinic and was prescribed risperidone and procyclidine over a six-month period. His psychiatric symptoms had been under control with no major complaints except for minor episodes of constipation managed at home with over-the-counter medications. Alongside the abdominal pain, he also complained of constipation and dark discoloration of his urine. There was also associated generalized weakness and tiredness. There was no significant family history of note.

Physical examination in the emergency department revealed a pulse of 100 beats per minute, blood pressure of 139/68 mmHg, temperature of 37.3 °C, and oxygen saturation of 99% on room air. Respiratory and cardiovascular examinations were unremarkable with a clear chest and normal heart sounds on auscultation. A gastrointestinal examination revealed a tense and distended abdomen with generalized tenderness but no signs of peritoneal inflammation. Upon auscultation, bowel sounds were reduced. On neurological examination, parkinsonism and specifically cogwheel rigidity were noticed in both upper limbs with no additional significant neurological findings.

**Figure 1:**
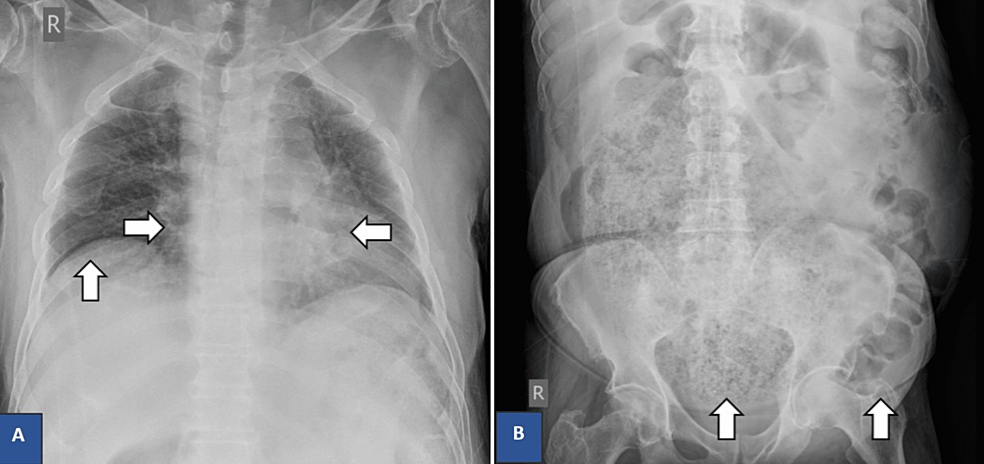
Plain radiography on day one of admission. A) chest radiography demonstrating enlargement of the cardiac silhouette and elevation of the right hemidiaphragm; B) abdomen radiography demonstrating massive dilation of bowel loops mainly involving the rectum and recto-sigmoid junction with impacted fecal material.

Laboratory blood tests demonstrated abnormalities such as leukocytosis, hypomagnesemia, and elevated creatine kinase levels (Table 1). Routine urinalysis showed no abnormalities. Plain radiography demonstrated massive dilatation of large bowel loops, fecal impaction, and a raised right hemidiaphragm (Figure 1). A CT scan of the brain was also performed which showed no evidence of any acute changes at the time of the study.

**Table 1:** Laboratory blood test results during the course of the admission

Laboratory test (normal values)	Day 1	Day 2	Day 3	Day 4	Day 5	Day 6	Day 7	Day 8
White blood cells (3.6-9.6 x10^9^/L)	11.7	10.8	11.8	13.9	14.1	16.2	14.7	13.9
Hemoglobin (12.0-14.5g/dL)	13.0	12.7	12.6	12.4	12.0	11.9	12.3	11.9
Urea (3.2-8.2 mmol/L)	6.3	7.3	6.4	6.8	8.5	13.1	22.6	24.6
Creatinine (40-66 µmol/L)	76	61	68	63	69	77	130	115
Sodium (132-146 mmol/L)	138	138	143	137	135	135	135	141
Potassium (3.5-5.5 mmol/L)	3.8	3.8	3.6	3.4	3.9	3.8	4.2	3.9
Calcium (2.15-2.50 mmol/L)	2.19	2.31	2.12	2.16	2.30	2.13	2.10	1.96
Magnesium (0.9-1.1 mmol/L)	0.7	0.9	0.8	0.8	0.9	0.7	0.8	0.9
Creatine Kinase (34-145 U/L)	1781	-	863	501	142	176	163	80
Procalcitonin (< 0.05 μg/L)	-	-	0.78	-	2.06	-	4.72	-
C-reactive protein (0-5 mg/L)	-	-	333	-	405	-	412	-
Lactic acid (< 2 mmol/L)	-	-	-	2.0	-	-	2.6	-

The patient was then admitted to a general medical ward with an initial impression of rhabdomyolysis and constipation. Upon review in the emergency department, the psychiatry service advised holding risperidone and procyclidine until his condition improved. He was managed conservatively with IV fluids, electrolyte replacement therapy, and laxatives for three days with no major improvement. The patient was not tolerating orally due to nausea and he did pass stool after enema application. On digital rectal examination, only small amounts of soft stool were noted. On the fourth day of admission, the patient complained of shortness of breath, worsening abdominal distention, and became hypoxic. He was started on supplemental oxygen via nasal cannula and a septic work-up was requested. Laboratory analysis revealed leukocytosis and elevated inflammatory markers. Blood and urine cultures revealed no growth and chest radiography did not show any infiltrates. During follow-up, the patient continued to remain constipated despite repeated enema applications.

**Figure 2:**
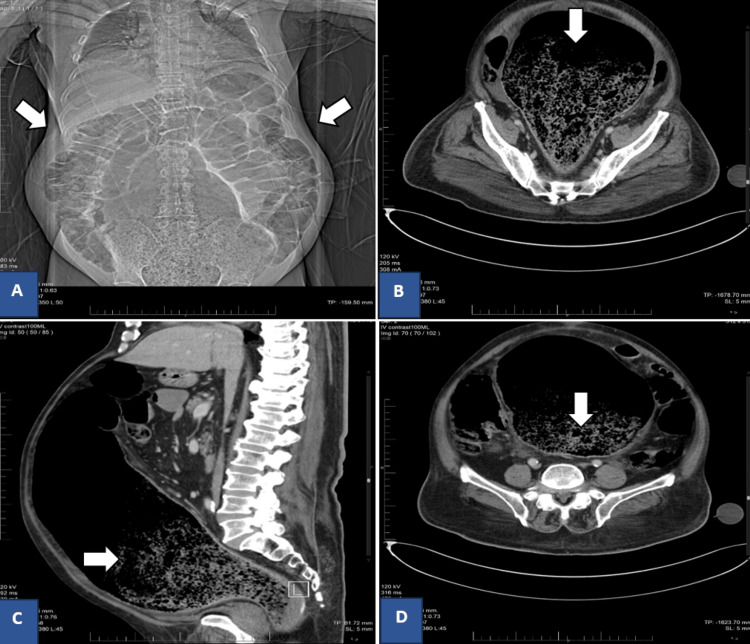
CT performed during admission. A) coronal view of the abdomen showing massive large bowel dilatation seen more prominently at the rectum; B, C, and D): axial and sagittal views redemonstrating the dilatation of large bowel with a maximum diameter of 18 cm at the level of the rectum and no visible transitional zone, mass, or region of mechanical obstruction.

The general surgical service was consulted and advised the insertion of a wide-bore nasogastric tube for drainage, starting on broad-spectrum antibiotics, and an urgent high-dose contrast-enhanced CT (CECT) scan of the abdomen. The CECT showed a significantly dilated fecally impacted rectum and sigmoid colon with the sigmoid colon measuring approximately 18 cm and the rectum measuring 6 cm in diameter (Figure 2). There were no areas of mechanical obstruction and the diagnosis of Ogilvie’s syndrome was made. The surgical team later advised conservative management including a nil per oral order, nasogastric tube for drainage, aggressive replacement of fluid and electrolytes, rectal tube insertion, and arranging a colonoscopy for decompression. In addition to that, the psychiatry service was consulted regarding antipsychotic medications and advised to continue keeping them on hold with reconsideration for restarting once the general condition improved. The patient continued to pass small amounts of stool with laxative use; however, abdominal girth did not improve after insertion of the rectal tube. The patient was later reviewed by the gastroenterology team and a decision was made to not proceed with colonoscopy due to the high risk of perforation and the general patient’s condition. Additionally, the team recommended starting the patient on neostigmine. IV neostigmine infusion was therefore started at a rate of 0.4 mg/hr with close cardiac monitoring.

The patient’s condition continued to deteriorate with laboratory analysis showing leukocytosis, deteriorating renal function, and hypocalcemia. Chest radiography showed a right-sided opacity with loss of cardio-phrenic angle. The impression of pleural effusion and hospital-acquired pneumonia was made. The patient was then started on IV meropenem and linezolid for a hospital-acquired infection. Despite best efforts, the patient went into cardiopulmonary arrest on the ninth day of admission and all resuscitative methods were unsuccessful.

## Discussion

Ogilvie’s syndrome or ACPO is a clinical entity characterized by dilatation of the colon in the absence of an anatomic lesion that leads to intestinal obstruction [9]. The syndrome was first described in 1948 by an English surgeon named Sir William Heneage Ogilvie [10]. He described the disorder as massive dilatation of the cecum and right hemicolon with extension to the rectum in the absence of mechanical or anatomical intestinal obstruction [10]. It is not possible to predict which patients will develop ACPO and there are no definite causes; however, many clinical conditions that increase the risk for Ogilvie’s syndrome have been identified. Advanced age, comorbidities associated with electrolyte disturbance or polypharmacy, neuropsychiatric disorders, and poor underlying functional status or immobility are all associated factors [2].

In schizophrenia, the predisposition to developing Ogilvie’s syndrome could be due to an associated neurodevelopmental abnormality of the enteric nervous system [11]. Although antipsychotic medications are recommended for managing the symptoms of schizophrenia and other psychotic disorders, their adverse effects can be clinically significant. Constipation due to gastrointestinal hypomotility is a common and serious adverse effect [12]. The antipsychotic medication clozapine works as an antagonist of cholinergic, histaminergic, and serotonergic receptors and is frequently involved in antipsychotic‐related gastrointestinal hypomotility [11]. Clozapine has a higher anticholinergic activity in comparison to risperidone [13]. Constipation is a common adverse effect of risperidone; however, paralytic ileus is rarely recorded as associated with it as described in the literature [11]. In our case, the patient was known to suffer from a psychiatric disorder and was treated with risperidone. Upon revision of the psychiatry outpatient clinical notes, it was evident that the patient did complain of constipation after initiation of risperidone.

Ogilvie’s syndrome can be managed conservatively; but, if left without recognition or treatment, it can lead to serious and fatal complications [1]. Perforated viscous and bowel ischemia are the most serious complications that need emergency surgical intervention [14]. A diagnosis of Ogilvie’s syndrome can be made after taking a detailed history from the patient, identifying characteristic symptoms, performing a thorough examination and clinical evaluation, and ruling out other conditions that cause similar presentations by utilizing multiple specialized tests. The characteristic feature of ACPO is the significant dilatation of the cecum and transverse colon in the absence of an abrupt transition point or mechanical intestinal obstruction [15]. However, a transition point may present gradually at or near the splenic flexure [14]. CT imaging usually provides an accurate measurement of the degree of bowel dilation in order to direct the decision for the needed intervention [16].

The initial management of Ogilvie’s syndrome involves treating the underlying condition and correcting any biochemical abnormalities [1]. Antibiotics can be administered empirically when there is a suspicion of bowel ischemia or perforation [17]. In the current case presentation, our patient was initially managed conservatively based on the finding of a dilated bowel in plain radiography. However, due to the failure of the conservative approach and CT imaging findings, the surgical team confirmed the diagnosis of Ogilvie’s syndrome.

When conservative approaches fail, neostigmine is indicated for the treatment of ACPO [18]. Neostigmine is an acetylcholinesterase inhibitor that increases intestinal contractility. Of those receiving neostigmine, 80% will express immediate colonic decompression with minimal recurrence in only 3-5% of cases [12]. A two-year retrospective comparative study was done on patients diagnosed with Ogilvie’s syndrome in an ICU in Turkey [18]. One of the two neostigmine protocols, the bolus dose or continuous infusion, was utilized in patients not responding to conservative management. The study concluded that different neostigmine protocols did not show any significant difference with regard to safety and effectiveness. In the literature, neostigmine infusion has been shown to be effective in refractory cases [12]. Another commonly used medication in ACPO is prucalopride which is a 5-hydroxytryptamine agonist that stimulates colonic motor activity [12]. If pharmacological therapies fail or are contraindicated, patients should be assessed for endoscopic decompression. The procedure is, however, technically difficult and needs an experienced specialist [2]. In this instance, decompression is achieved through colonoscopy without bowel preparation [2]. Additionally, in severe cases, surgical or fluoroscopy-assisted cecostomy is necessary or a percutaneous endoscopic colostomy is needed [9].

In our case, the patient was not offered endoscopic decompression or colonoscopy due to the high risk of cecal perforation and due to his poor general condition. Therefore, an infusion of neostigmine was commenced in addition to close cardiac monitoring. Unfortunately, The patient’s condition rapidly deteriorated and he went into cardiopulmonary arrest. Fever and severe abdominal pain with or without signs of peritonitis are alarming findings suggestive of intestinal ischemia and perforation [2]. Additionally, sepsis should be considered in cases of peritonitis with signs of shock [2]. Upon deterioration, our patient had features including abdominal tenderness, fever, and leukocytosis, alongside cecal dilatation of more than 12 cm. These features may indicate colonic ischemia or perforation contributing to a rapid deterioration. Given the high mortality rate, strong levels of suspicion and early diagnosis in cases of mental and psychiatric conditions may prevent delays in the treatment and result in lower morbidity and mortality. Statistically, the mortality rate is as low as 15% if the diagnosis is made early, while it increases to 36-44% in late diagnosis [2].

## Conclusions

Ogilvie’s syndrome is a rare cause of bowel obstruction and is considered a diagnosis of exclusion. Our case demonstrated the high risk of developing Ogilvie’s syndrome in an elderly patient known to be on the antipsychotic agent risperidone. Antipsychotic-related gut hypomotility, specifically clozapine-related gut hypomotility, has been documented in the literature. On the other hand, Ogilvie’s syndrome association with risperidone is rarely reported. Additional factors, such as in our case, include advanced age and electrolyte imbalances. The prognosis is dependent on the patient’s age, severity of the predisposing disease, maximal diameter of the colon, and duration of colonic distention. Cases may be managed conservatively, with direct pharmacological intervention, endoscopically, or surgically. Timely identification is of utmost importance in the diagnosis and management of patients with Ogilvie’s syndrome in order to avoid the high rates of morbidity and mortality associated with this condition.
